# Clinical complications of G6PD deficiency in Latin American and Caribbean populations: systematic review and implications for malaria elimination programmes

**DOI:** 10.1186/1475-2875-13-70

**Published:** 2014-02-25

**Authors:** Wuelton M Monteiro, Gabriel P Franca, Gisely C Melo, Amanda LM Queiroz, Marcelo Brito, Henry M Peixoto, Maria Regina F Oliveira, Gustavo AS Romero, Quique Bassat, Marcus VG Lacerda

**Affiliations:** 1 Fundação de Medicina Tropical Dr. Heitor Vieira Dourado (FMT-HVD), Av. Pedro Teixeira, 25, Dom Pedro, Manaus, AM 69040-000, Brazil; 2 Escola Superior de Ciências da Saúde, Universidade do Estado do Amazonas (ESA-UEA), Manaus, AM, Brazil; 3 Faculdade de Medicina, Universidade de Brasília (UnB), Brasília, DF, Brazil; 4 National Institute for Science and Technology for Health Technology Assessment (IATS/CNPq), Porto Alegre, RS, Brazil; 5 Barcelona Centre for International Health Research (CRESIB, Hospital Clínic-Universitat de Barcelona), Barcelona, Spain

**Keywords:** Glucose-6-phosphate dehydrogenase deficiency, Primaquine, Haemolysis, Malaria, *Plasmodium vivax*

## Abstract

**Background:**

Although G6PDd individuals are generally asymptomatic throughout their life, the clinical burden of this genetic condition includes a range of haematological conditions, including acute haemolytic anaemia (AHA), neonatal jaundice (NNJ) and chronic non-sphaerocytic anaemia (CNSA). In Latin America (LA), the huge knowledge gap regarding G6PDd is related to the scarce understanding of the burden of clinical manifestation underlying G6PDd carriage. The aim of this work was to study the clinical significance of G6PDd in LA and the Caribbean region through a systematic review.

**Methods:**

A systematic search of the published literature was undertaken in August 2013. Bibliographies of manuscripts were also searched and additional references were identified. Only original research was included. All study designs were included, as long as any clinical information was present. Studies were eligible for inclusion if they reported clinical information from populations living in LA or Caribbean countries or about migrants from these countries living in countries outside this continent.

**Results:**

The Medline search generated 487 papers, and the LILACS search identified 140 papers. After applying the inclusion criteria, 100 original papers with any clinical information on G6PDd in LA were retrieved. Additionally, 16 articles were included after reading the references from these papers. These 116 articles reported data from 18 LA and Caribbean countries. The major clinical manifestations reported from LA countries were those related to AHA, namely drug-induced haemolysis. Most of the published works regarding drug-induced haemolysis in LA referred to haemolytic crises in *P. vivax* malaria patients during the course of the treatment with primaquine (PQ). Favism, infection-induced haemolysis, NNJ and CNSA appear to play only a minor public health role in this continent.

**Conclusion:**

Haemolysis in patients using PQ seems to be the major clinical manifestation of G6PDd in LA and contributes to the morbidity of *P. vivax* infection in this continent, although the low number of reported cases, which could be linked to under-reporting of complications. These results support the need for better strategies to diagnose and manage G6PDd in malaria field conditions. Additionally, Malaria Control Programmes in LA should not overlook this condition in their national guidelines.

## Background

Glucose-6-phosphate dehydrogenase (G6PD) is an important enzyme that catalyses the first reaction in the pentose-phosphate pathway. Within the erythrocyte, G6PD is the sole source of enzymatic activity that protects against the build-up of super-radicals and, thus, oxidative stress [1,2]. G6PD deficiency (G6PDd) is an X-linked, hereditary genetic defect caused by mutations in the G6PD gene, resulting in protein variants with different levels of enzymatic activity that are associated with a wide range of biochemical and clinical phenotypes [2]. G6PDd heterozygosity and hemizygosity have been associated with approximately 50% protection against severe *Plasmodium falciparum* malaria [3,4]. Therefore, the high prevalence rates of G6PDd in many parts of the world can most likely be accounted for by the selection pressure exerted by malaria. The current estimated prevalence of G6PDd across malaria-endemic countries is approximately 8%, which corresponds to *circa* 350 million affected individuals in these countries. The lowest prevalence is known to occur in the Americas, while the highest prevalence is observed in tropical Africa, Middle East and the Mediterranean basin [5].

Although G6PDd individuals are generally asymptomatic throughout their life, the clinical burden of this genetic condition includes a range of haematological conditions, including acute haemolytic anaemia (AHA), neonatal jaundice (NNJ) and chronic non-sphaerocytic anaemia (CNSA) [1,2]. Because AHA is the most common manifestation of G6PDd, it is critical to understand how such episodes can be prevented. In G6PDd individuals, these complications are usually triggered by the advent of specific infections or the intake of certain food products, typically fava beans. As such, this inherited disorder is also known by the alternative name “favism”. Complications can also be triggered by drugs, the prime example of which is the anti-malarial primaquine (PQ). The prevention of food-induced haemolytic crises, such as in favism, should theoretically be fully preventable by avoiding the triggering foods. In contrast, the prevention of infection-induced haemolysis is obviously more difficult. In most cases, the prevention of drug-induced haemolysis is possible by choosing alternative drugs, but this may be difficult when no alternatives are available. This difficulty becomes particularly relevant when attempting to treat *Plasmodium vivax* infections, as radical cures, including the specific treatment of the latent hepatic stages that are mainly responsible for subsequent relapses after the first clinical episode, require the use of PQ for 7 to 14 days. In some individuals, this treatment can lead to life-threatening anaemia and acute renal failure [6]. In these cases, administration of a lower dosage for a longer time is the recommended approach, but only in those patients with mild-to-moderate enzymatic activity. In such patients, haemolysis will still occur, but under appropriate surveillance, it will be of an acceptably mild degree [1]. Importantly, the use of PQ in patients with a low enzymatic activity is contraindicated. It is critical to understand that the risk is variant-dependent and dose-dependent and cumulative. PQ is also the only currently available drug that is effective against *P. falciparum* stage V gametocytes. The potentially hazardous effect of this drug in G6PDd individuals jeopardizes any population-wide effort to reduce malaria transmission via drugs such as PQ due to the impossibility of performing rapid population-wide screening for the deficiency and the hazards associated with the blind administration of such drugs [7]. More recently, the World Health Organization has recommended 0.25 mg/kg of PQ in a single dose for patients with *P. falciparum*, as this dose is efficacious as a gametocytocidal drug without the risks of inducing haemolysis in G6PD patients [8].

A thorough understanding of the clinical burden related to G6PDd could be of great value for understanding whether particular drug regimens could be used safely at a population level with or without prior knowledge of the individuals’ G6PD status, or whether the development of field-deployable and electricity-independent rapid diagnostic tests, resembling those that already exist for malaria diagnosis, is indispensable. Some authors have recently proposed maps of the G6PDd global distribution, including LA countries, highlighting the small amount of published works regarding this deficiency in this continent [5,9]. Large swathes of the American malaria endemic areas were predicted to have median G6PDd frequencies ≤1% (40.8% land area), with G6PDd being virtually absent from northern Mexico, Costa Rica, Peru, Bolivia, and much of Argentina. The prevalence increased towards coastal regions, peaking in Venezuela, where the majority of the continent’s predictions of >5% prevalence were located [5]. Only a limited repertoire of variants was observed across LA countries, and surveys indicated relatively low genetic heterogeneity, which was predominated by the African variant [10]. Moreover, in the Americas, the huge knowledge gap is related to the scarce understanding of the burden of clinical manifestation underlying G6PDd carriage. The aim of this work was to study the clinical significance of G6PDd in LA and the Caribbean region through the systematic review of available published data.

## Methods

### Geographic coverage of the study

South America, Central America, Mexico and the Caribbean area constitute a vast territory formed by 33 independent countries and 20 autonomous or semi-autonomous territories. They cover a territorial area of over 20 million km^2^, which included a population of approximately 580 million inhabitants in 2008. With regards to ethnicity, in some countries, mainly Chile, Argentina and Uruguay, white people of European origin predominate. In others, such as Venezuela, Brazil, Paraguay, Nicaragua and Colombia, white immigrants mixed noticeably with the Amerindians. Finally, in some countries, such as Mexico, Guatemala, El Salvador, Honduras, Panama, Peru, Bolivia and Ecuador, the Amerindians still constitute a numerically important portion of the population. Additionally, the continent has received multiple waves of African immigrants, especially to the Caribbean area.

### Systematic review

Potentially relevant papers in all languages were accessed from Medline and LILACS to review their full texts. A broad free text search using the combination of Medical Subject Heading (MeSH) terms and keywords presented in Table 1 was utilized.

**Table 1:** Keywords and MESH headings used for literature searches

**Database**	**Search terms**
Medline	(favism OR glucosephosphate dehydrogenase OR glucosephosphate dehydrogenase deficiency OR G6PD OR g6pd deficiency OR G-6-PD OR g-6-pd deficiency OR glucose-6-phosphate dehydrogenase OR glucose-6-phosphate dehydrogenase deficiency) AND (Antilles OR Latin America OR South America OR Central America OR Caribbean OR Anguilla OR Antigua OR Aruba OR Barbuda OR Argentina OR Bahamas OR Barbados OR Belize OR Bolivia OR Brazil OR Chile OR Colombia OR Costa Rica OR Dominica OR Dominican Republic OR Ecuador OR El Salvador OR Grenada OR Grenadines OR Guadeloupe OR Guatemala OR Guyana OR Haiti OR Honduras OR Jamaica OR Martinique OR Mexico OR Montserrat OR Nevis OR Nicaragua OR Panama OR Paraguay OR Peru OR Puerto Rico OR Saint Kitts OR Saint Lucia OR Saint Vincent OR Suriname OR Surinam OR Trinidad OR Tobago OR Uruguay OR Venezuela) [MeSH]
LILACS	(favism OR glucosephosphate dehydrogenase OR glucosephosphate dehydrogenase deficiency OR g6pd OR g6pd deficiency OR g-6-pd OR g-6-pd deficiency OR glucose-6-phosphate dehydrogenase OR glucose-6-phosphate dehydrogenase deficiency)

Only original research of all study designs (clinical trials, cohort studies, case–control studies, cross-sectional studies, case series and case reports) was included, as long as any clinical information was present. Additional articles were obtained through citation tracking of reviews, opinion articles and original papers. Studies were eligible for inclusion if they reported clinical information from populations living in LA or Caribbean countries or about migrants from these countries living in countries outside this continent. An individual was considered to have G6PDd if they presented with any positive diagnostic test, such as DNA analysis, enzyme activity assay, NADPH fluorescent spot, brilliant cresyl blue, gel electrophoresis, methylene reduction test or phenazine methosulphate-3-(4,5-dimethylthiazal-2-yl)-2,5-diphenyltetrazolim bromide. In this study, clinical information refers to G6PDd manifestations, such as AHA, NNJ and CNSA, as well as G6PDd coexisting with other genetic disorders and association of G6PDd with susceptibility or clinical presentations of metabolic disorders or other infectious diseases than malaria. In this study, a history of jaundice or red cell transfusion after drug therapy was used as a *proxy* for AHA. To identify relevant papers, the titles, abstracts and the full texts of the studies were examined by two independent reviewers. The data were directly extracted from the full-length articles to structured table and figures containing all the descriptive variables and relevant outcomes.

## Results

**Figure 1:**
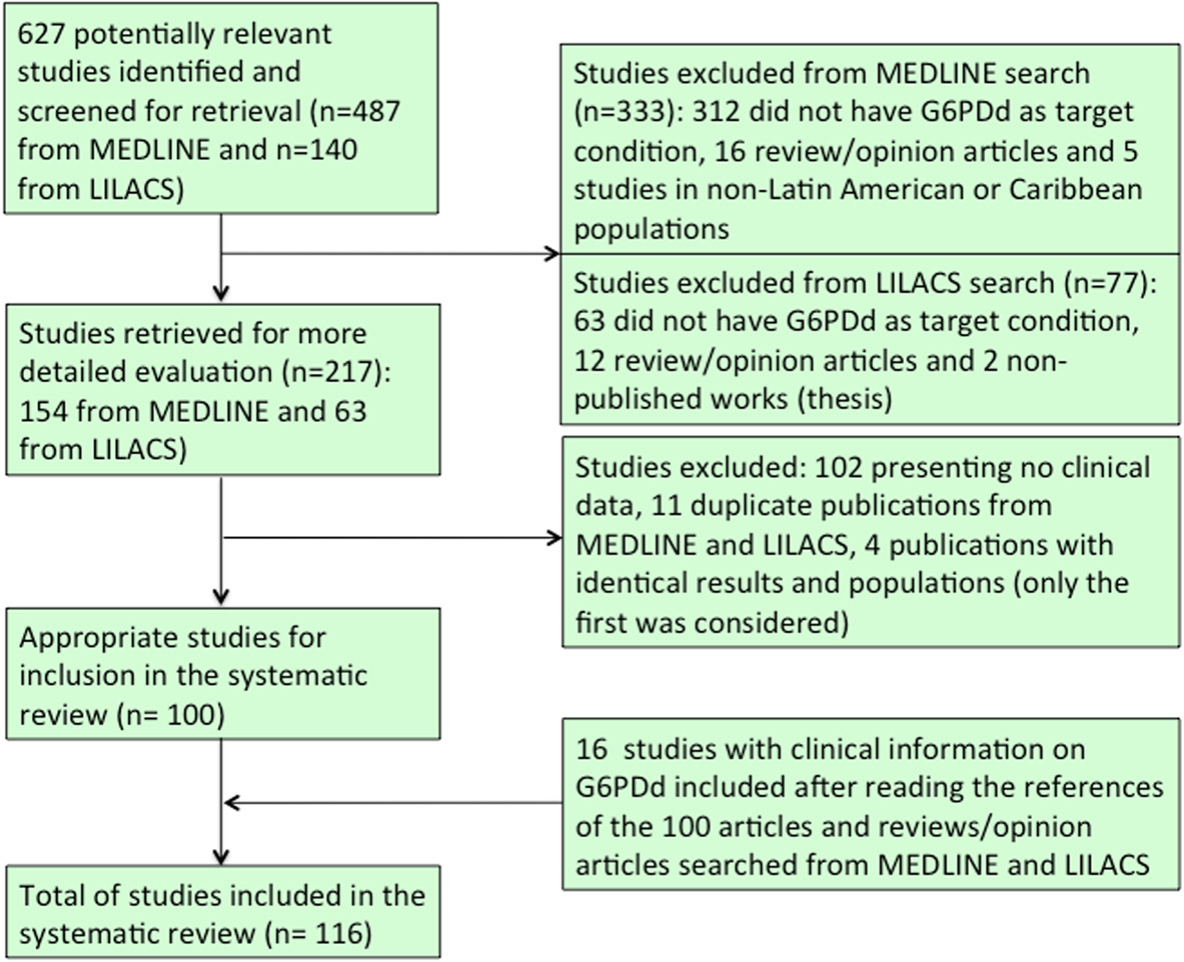
Flow chart of inclusion of studies reporting clinical information on G6PD deficiency in Latin America and Caribbean countries.

The Medline search generated 487 papers, and the LILACS search generated 140 papers. After applying the inclusion criteria to those papers, 100 original papers with any clinical information on G6PDd in LA were retrieved. Additionally, 16 articles were included after reading the references of the 100 articles and reviews/ opinion articles that were obtained from the Medline and LILACS searches (Figure 1). These 116 articles reported data from 18 LA countries.

### Clinical manifestations of G6PDd in LA and Caribbean populations

#### Acute haemolytic anaemia (AHA)

**Figure 2:**
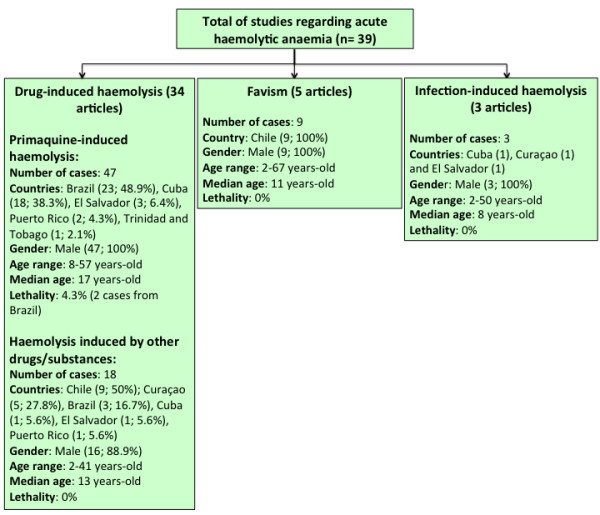
Major findings on acute haemolytic anaemia in Latin America and the Caribbean countries.

Additional file 1 and Figure 2 summarize the AHA findings. Data on AHA were reported from 39 publications, including 34 regarding drug-induced haemolysis, five favism and three infection-induced haemolysis. Some included publications presented two or more clinically relevant G6PDd manifestations. A total of 107 characterized cases of AHA in LA were found. Out of these cases, for 30 (28.0%), it was not possible to identify the triggering cause. Drug-induced haemolysis was responsible for 65 cases (60.7%), with PQ responsible for 47 (43.9%) and other drugs/substances for 18 (16.8%) cases. Nine (8.4%) cases of AHA were due to favism, and three (2.8%) cases were attributed to infection-induced haemolysis.

Figure 2 shows that the AHA cases were mostly reported in men (105/107; 98.1%). PQ-induced haemolysis, favism and infection-induced haemolysis were reported uniquely in males. Only 2 cases of haemolysis (one triggered by naphthalene poisoning and one by salicylates), were registered in females. Although AHA was observed in all ages, most of the cases were reported in the young population, with the median age ranging from 8 to 17 years for infection-induced haemolysis and PQ-induced haemolysis, respectively. Case fatalities were reported only among PQ-induced haemolysis cases, with a 4.3% rate.

Among the 107 cases of AHA, it was possible to identify the ethnicity for only 24 (22.4%) subjects, being 8 (17.0%) presenting PQ-induced haemolysis, 8 with haemolysis induced by other drugs/substances (44.4%), 6 with favism (66.7%) and 2 (66.7%) with infection-induced haemolysis. PQ-induced haemolysis cases predominated among admixed individuals (7/8; 87.5%). Haemolysis induced by other drugs/substances (6/8; 75%) and infection-induced haemolysis (2/2;100%) predominated among black individuals. Favism was recorded in white individuals only (6/6; 100%).

**Figure 3:**
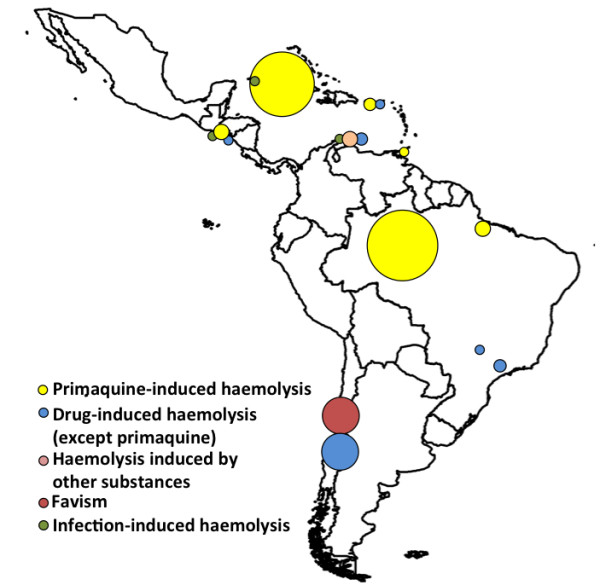
**Geographic distribution of the acute haemolytic anaemia cases, according to triggering causes, in Latin American and Caribbean countries.** The circles are proportional to the number of cases at the site.

Figure 3 shows the geographic distribution of the AHA cases in which it was possible to identify the triggering haemolytic cause. The highest number of cases was reported in Brazil (26; 33.8%), followed by Cuba (19 cases; 26.7%) and Chile (18 cases; 23.4%). Primaquine-induced haemolysis was reported in Brazil (23 cases; 48.9%), Cuba (18 cases; 38.3%), El Salvador (three cases; 6.4%), Puerto Rico (two cases; 4.3%), and Trinidad and Tobago (one case; 2.1%).

### Drug-induced haemolysis

Some cross-sectional studies with a recall approach showed an absence of signs of haemolysis among the G6PD deficient population [11-14]. One study showed that patients who received blood donated by G6PDd individuals did not develop haemolysis, even though some were using drugs that could potentially trigger this complication [15]. Some studies showed a higher frequency of previous history of jaundice in G6PDd in relation to non-G6PDd individuals [16], or a higher frequency of G6PDd in patients with history of acute haemolytic crises in comparison with the general population [17]. However, haemolysis caused by G6PDd was generally mild [16,18,19].

Reports of occasional moderate haemolytic anaemia were reported in Brazil [20], Costa Rica [21] and Mexico [18,22,23], with poor clinical descriptions and no identification of the stressor. No complication was reported in patients using the single dose of PQ (0.75 mg/kg) for *P. falciparum* gametocytes. In Mexico [24], Ecuador [25] and Chile [26], the frequency of G6PDd in patients with haemolytic anaemia was 30.3, 8.6 and 39.5%, respectively, but the authors did not detail the haemolysis-triggering causes for all patients. Haemolytic episodes in G6PD deficient subjects related to naphthalene intoxication, use of salicylates, transfusion of G6PDd blood into recipients with leprosy under sulpha drug therapy and excessive intake of rum or wine were reported in Curaçao [27]. In LA countries, there were reports of drug-induced haemolytic episodes triggered by nalidixic acid [28], chloramphenicol [26], aspirin [26,29], sulphadiazine [26,30,31], sulphazoxazole plus nitrofurantoin [32] and acetaminosalol [26]. In Brazil, G6PDd did not seem to be associated with a higher risk of haemolysis in patients who were being treated with sulphone [33], ketoconazole [34], or who were under spinal anaesthesia with bupivacaine or general intravenous anaesthesia with propofol [35]. In Saint Lucia, G6PD-deficient children infected with *S. mansoni* were given a single intramuscular dose of hycanthone, but their subsequent serial haematocrit and reticulocyte counts showed no evidence of haemolysis [36].

In 1970, three patients from El Salvador who presented with PQ-induced haemolysis were confirmed as G6PD deficient, and one of these patients required an exchange transfusion [29]. Two cases of PQ-induced haemolysis were reported from Puerto Rico [32]. Haemolysis appeared in 87.5% of 16 G6PD-deficient patients from Cuba. This effect occurred mainly on the fifth day of treatment, after the administration of 105 mg of PQ [37]. In Trinidad, the management of two imported cerebral malaria cases was complicated by their G6PD-deficient status, with the occurrence of blackwater fever, cerebral manifestations, renal impairment, hyperglycaemia and thrombocytopaenia [38]. In Cuba, there were reports of haemolysis in six of eight (87.5%) G6PDd patients treated with PQ, three of whom could not finish the treatment [39]. Treatment discontinuation was also described in cases from Brazil, where three G6PDd patients with vivax malaria in a chloroquine plus PQ regimen presented with AHA [40]. In a similar regimen, 18 G6PD-deficient patients presented with AHA that required red blood cell transfusions and finally developed acute renal failure [41]. In the Brazilian Amazon, G6PDd was associated with a considerably higher risk of malaria-related transfusions likely triggered by the treatment for malarial infection [42,43]. In Manaus, an autopsy series of deceased patients with confirmed *P. vivax* infection could only attribute the cause of death to PQ-induced haemolysis, demonstrating the lethal potential of this condition [44].

The use of chloroquine alone did not trigger haemolysis in 8 G6PD-deficient subjects [45].

### Favism

The search identified five publications from Chile that reported a total of nine cases of favism in LA [26,46-49]. All cases occurred in males, and most were in children. In three cases, blood transfusions were needed, and one individual evolved to acute renal failure. There was no record of case fatalities.

### Infection-induced haemolysis

In Cuba, recurrent viral and bacterial infections were indicated as possible causes of haemolysis in an 8-year-old boy [19]. In Curaçao, one case of haemolytic anaemia in a G6PD-deficient subject was related to a febrile non-defined viral infection [27]. In El Salvador, one episode of haemolysis was reported in a patient with an infection without defined aetiology, but the stressor was not characterized because the subject was taking aspirin [29]. A clear aetiological trigger could not be confirmed in any of these cases.

### Neonatal jaundice (NNJ)

Neonatal jaundice was reported in 30 original articles. The frequency of NNJ in G6PD-deficient new-borns ranged from 38.5 to 100% [50-56]. However, excluding other causes, the aetiology of jaundice was attributed to G6PDd in 5 [57] to 15% [58] of the new-borns. On the other hand, G6PDd was detected in 3.4% of male new-borns with NNJ in the absence of foetal-maternal incompatibility in Costa Rica [59] and in 69.6% of a group of neonates who had unexplained moderate to severe jaundice [60]. Four studies reported no severe complications in G6PD-deficient new-borns with NNJ [53,54,56,59], while three works described the need for exchange transfusions [50,51,55], three described the occurrence of kernicterus [51,60,61], and two described fatal outcomes [29,60].

Some cross-sectional studies have demonstrated that NNJ cannot be solely attributed to G6PDd [62] or a lack of association between G6PDd and NNJ [24,63-67]. In Trinidad, a similarity between the frequencies of enzyme defects in normal children and those with cerebral palsy suggested that neonatal jaundice associated with G6PDd may be a factor of little significance in the development of kernicterus and subsequent cerebral palsy, even in a community with a high prevalence of this enzyme deficiency [68]. In a prospective study from Brazil, G6PDd did not appear as a risk factor for moderate hyperbilirubinaemia [69], and in a case–control study in the same country, G6PDd was not found to be related to severe hyperbilirubinaemia [70]. Other reports have found contradictory results about the relationship between G6PDd and NNJ in LA, suggesting that there was a positive association between G6PDd and NNJ [71-77]. In a work from Mexico, the prevalence of NNJ was only linked to G6PDd in male babies [52].

### Chronic non-sphaerocytic anaemia (CNSA)

Data concerning the association between G6PDd and anaemia, including chronic non-sphaerocytic anaemia, were presented in 18 articles. Only 8 defined cases of CNSA were recorded from Latin America, with 2 from Brazil [78,79], 2 from Chile [80] and 1 each from Cuba [81], Costa Rica [82], Mexico [24] and Puerto Rico [32]. However, in general, these publications had poor clinical descriptions, as they were primarily aimed at characterising the enzyme. Of these cases, those from Chile had more detailed clinical data and highlighted persistent anaemia with the need for blood transfusions, reticulocytosis, jaundice and splenomegaly [80]. Cholelithiasis is a complication that can follow CNSA, but in Brazil, there was no association between the need for cholecystectomy and G6PDd [83]. In a study from Brazil [84] and in another from Colombia [85], G6PD-deficient subjects showed a lower level of haemoglobin than the normal individuals.

Most of the published reports describing haematological parameters in LA, which covered several distinct geographical areas, showed similar haematimetric indexes in G6PDd individuals and non-deficient ones [86-92]. In Brazil, a low incidence of anaemia conferred no important haematological changes in individuals in which G6PDd was found [93].

### Coexistence of G6PDd with other genetic disorders

The coexistence of G6PDd with other genetic disorders was reported in 14 articles. In Argentina, beta-thalassemia was detected in 4.8% of the G6PD-deficient subjects [94], and the concurrence of haemoglobin S and congenital sphaerocytosis with G6PDd was detected in 4 and 2% of the subjects, respectively [50]. In Brazil, haemoglobin S was detected in 11.1% of the G6PD-deficient individuals [95]. In Costa Rica, the frequency of G6PDd among male HbAS carriers ranged from 2.3 [96] to 15% [97]. However, controlled studies have shown that G6PDd is independent from the occurrence of abnormal haemoglobin [27,98-100], HbS or HbC [101] sickle cell trait [102] and sickle cell disease [103]. In Jamaica, difference in G6PD status did not affect the total haemoglobin concentration, reticulocyte count, unconjugated serum bilirubin, Hb F concentration, plasma haemoglobin concentration and frequencies of clinical severity and of leg ulceration in patients with sickle cell disease [104]. In Brazil, G6PDd was absent in nine children with clinical diagnosis of glycogenoses [105].

### G6PDd and malaria susceptibility

An association between G6PDd and malaria susceptibility was reported in 8 articles. In a traditionally malaria-endemic area of South-eastern Brazil, the frequency of G6PDd and average G6PD activity was similar between the groups with and without a history of malaria [106]. *Plasmodium falciparum* infections were recorded at the same proportion in G6PDd and normal individuals in the Southern Brazilian Amazon [107]. In Brazil, an absence of association between G6PD phenotypes and the number of previous episodes of malaria in men was reported [108]. However, in Ecuador, G6PDd prevalence was higher in provinces that were non-endemic for malaria, compared to endemic provinces, suggesting an ecological association between G6PDd and some degree of protection against *P. falciparum*[109]. Colombian individuals with a complete G6PD deficiency had a lower density of *P. falciparum* parasitaemia than persons without this condition [110]. In Colombia, the mean G6PD activity was lower, and G6PDd was more prevalent among men without *P. vivax* malaria than in those presenting with the disease [111]. In the Brazilian Amazon, where *P. vivax* predominates, G6PDd individuals were less likely to report the occurrence of malaria episodes after adjusting for age [42,43]. In the same area, the protective effect was related to the enzymatic activity, with carriers of the African variant presenting an 88% reduction and carriers of the Mediterranean variant presenting a 99% lower risk compared to non-deficient individuals [43].

### G6PDd and metabolic disorders

The presence of metabolic disorders in G6PDd patients was investigated in three articles. Significantly lower insulin levels were observed for G6PDd Brazilian men compared to the controls in both the intravenous and oral tolerance tests [112]. In Mexico, two diabetic G6PDd individuals did not show cataracts, whereas cataracts were identified in six other diabetic patients [113]. In Brazil, the mean cortisol levels observed in the first hour after ACTH stimulation in the G6PD-deficient patients were significantly lower than in the control group [114].

### Other findings

Other findings were mentioned in 10 original articles. A significant increase in methaemoglobinaemia was observed following oral therapy with PQ in patients with *P. vivax* presenting G6PDd [115,116]. Additionally, in Colombia, an association between haemolysis and physical exercise was observed in individuals with reduced G6PD activity [117]. In Brazil, G6PDd was suggested to contribute to haemolysis in patients with the viscerocutaneous form of loxoscelism, a condition produced by the bite of the recluse spiders of genus *Loxosceles*[118]. In Brazil, there have been reports of recurrent infections in children diagnosed with G6PDd [119,120]. In contrast, increased rates of urinary tract infections and neonatal jaundice were not substantiated among pregnant women from Puerto Rico, Dominican Republic, Mexico and other Caribbean areas living in the United States. However, the same authors reported increased rates of abortions, low-birth-weight infants and puerperal drops in red cell volumes in this population [64]. Transfusions of G6PDd blood that has been typed as the African variant seems to be safe, as a study carried out in Brazil had no reports of major haemolysis in recipients [121]. In Brazil, there was a lack of association between G6PDd and *S. mansoni*[122] and leprosy susceptibility [123].

## Discussion

Mapping studies have demonstrated that *P. vivax* is more widely distributed than *P. falciparum* and that this species is a potential cause of morbidity and mortality amongst the 2.85 billion people living at risk of infection, most of whom live in Central and South East Asia and LA [124]. In LA and the Caribbean, most of the countries remain endemic for malaria. Only Chile, Uruguay, Cuba, Bahamas, Jamaica and other small countries in the Caribbean are now considered malaria-free. Mexico, Haiti, Costa Rica, El Salvador, Panama, Argentina and Paraguay are classified as ‘malaria-eliminating’ countries. The others are still in phases of control [125]. The predominance of vivax malaria in these countries is especially relevant because the radical cure of *P. vivax* infections requires the use of PQ, which can lead to acute intravascular haemolysis in G6PDd individuals, resulting in severe anaemia and acute renal failure [6]. The public health consequences of this condition deserve special attention due to the impossibility of using PQ in regions where there is a high prevalence of this deficiency, further hampering transmission control efforts for this parasite species [7]. Despite the clinical and epidemiological significance of the interaction between G6PDd and malaria, the extent of its clinical consequences has not been properly measured in LA populations. In countries such as Brazil, where PQ is systematically prescribed at a dose of 0.5 mg/kg/day for 7 days with chloroquine to all patients with microscopic confirmation of vivax malaria, the cumulative risk of adverse events in G6PDd patients could be even more relevant.

The most commonly reported clinical manifestations reported from LA countries were those related to AHA, namely drug-induced haemolysis. In patients with haemolytic anaemia, the reported frequency of G6PDd could be higher than 30% [24,26,126]. Favism and infection-induced haemolysis appear to play only a minor public health role in this continent. In general, in population-based studies carried out in regions where malaria is not endemic, individuals did not show a great risk of developing G6PDd-related haemolysis in their lifetimes [11-14,16,18,19], despite the establishment of an association between jaundice or history of haemolysis with G6PDd by some authors [16,17]. Another important finding in this context is the difficulty in linking G6PDd-related haemolysis with a specific stressor drug [20,22,23,25,26,126,127]. As reported, only a few sporadic haemolytic episodes were triggered by nalidixic acid, chloramphenicol, aspirin, salicylates, sulfadiazine, sulphazoxazole and nitrofurantoin and by naphthalene poisoning. These drugs or their metabolites are already known to be haemolysis triggers, most likely due to the production of free radicals, which in turn oxidize glutathione (GSH) and eventually lead to cell damage [128].

Most of the published works regarding drug-induced haemolysis in LA referred to haemolytic crises in patients diagnosed with *P. vivax* malaria during the course of the treatment with PQ. Reports of the need for red cell transfusions were common in these cases. In this continent, complications such as severe anaemia and renal failure also seem to be common and are of public health concern. Likewise, lethality was reported in the Brazilian Amazon, highlighting the impact on the local public health systems. Most strikingly, the results of two studies showed that the frequency of haemolysis in G6PDd patients was almost 90% [37,39]. Specifically, the most feared complication of PQ administration is the precipitation of haemolysis in G6PDd individuals. Other major PQ side effects that were identified in this review included methaemoglobinaemia, which again, mostly occurred among G6PDd patients. In this context, discontinuation of PQ treatment was observed in some countries, strongly suggesting that G6PDd in LA is hampering the transmission control efforts for *P. vivax*.

American countries contribute to 4.5% of the G6PDd male population from malaria endemic countries, corresponding to an estimated 10 million males, according to population data from 2010 [5]. Based on this estimation of G6PDd males living at risk of vivax malaria and consequently, of PQ-induced haemolysis, the number of AHA cases most likely is deeply underestimated, considering the literature data presented here. Firstly, this bias is likely related to a failure to recognize or properly diagnose AHA episodes among the malaria-affected population. In LA, malaria diagnosis and treatment are mostly made by technicians and only a few cases, especially complicated cases, are referred to tertiary-care centres. Accordingly, there is a lack of systematic diagnosis and surveillance of PQ-induced haemolysis in these countries, possibly leading to a significant proportion of under diagnosed cases. Secondarily, this lack of attention could lead to publication bias, as not many research groups are working in units linked to the tertiary health services located in malaria endemic areas. Therefore, published studies may not be truly representative of all of the information about G6PDd in LA, thus introducing a limitation to this systematic review.

This review points to the need for systematic testing of G6PDd in LA malaria endemic countries. Only with this result would it be possible to appropriately guide treatment, adjusting the PQ dosage or even contraindicating the drug, depending on the enzymatic activity of G6PD. The exposed population is extremely large in many endemic areas, and testing for G6PD status in all individuals would be a costly exercise, the cost-effectiveness of which remains to be evaluated. Furthermore, only a small number of investigations estimating G6PDd prevalence and the relative frequencies of the different genetic variants were carried out in LA malaria-endemic areas. Studies developed in the Brazilian Amazon found an estimated prevalence of 4%, with the African and Mediterranean variants predominating [43]. This review found that in publications from LA, PQ-induced haemolysis was only reported among males. This observation is in agreement with previous reports that clinically relevant G6PDd is much more common in males than in females because of the genetic nature of this condition. This result is of great significance for public health programmes because hemizygous females carrying G6PDd variants seem to be at reduced risk of PQ-induced haemolysis. The distinctive regional-specific character of the G6PDd clinical picture may help in the development of more targeted diagnostic approaches and PQ therapeutic strategies. In this regard, only testing the male population for G6PDd may be a more efficient and cheaper measure for minimising the risk of clinically relevant haemolysis after PQ administration in LA malaria-endemic areas.

A sound analysis on the role of the ethnicity in the geospatial distribution of G6PDd-related events was greatly impaired by the lack of information on ethnicity background for research subjects. It is known that global prevalence of G6PDd, and probably of its clinical manifestations, is geographically correlated with areas inhabited by populations historically exposed to endemic malaria, including Africa, Middle East, Mediterranean Europe and South-East Asia [5]. In this work, PQ-induced haemolysis predominated among admixed individuals, reflecting probably the ethnic composition of population living in *P. vivax* malaria endemic areas, and consequently the areas where PQ is prescribed, namely the Amazon. Favism was recorded in white individuals, in agreement with the literature that points that this is a condition typical of males of Mediterranean descent.

As previously demonstrated, the combination of G6PDd with the sickle cell trait was no more frequent than that expected by chance [129]. Several studies have shown that there are no significant differences in a variety of clinical and haematologic parameters between two otherwise comparable groups of patients with sickle cell anaemia, those with and without G6PD deficiency [130,131]. However, it must be borne in mind that acute intravascular haemolysis superimposed with chronic severe extravascular haemolysis is an added risk within this association. The combination of G6PDd with the β-thalassaemia trait has been found to cause a significant increase in mean corpuscular volume [132], although it remains below the normal range.

In LA, there are results suggesting that G6PDd prevalence could be a marker of the selective pressure exerted by malaria. The geographic distribution of G6PDd suggests that some polymorphisms confer resistance to falciparum malaria [5]. This phenomenon has been investigated mainly for the African variant, which has been shown to confer protection against lethal falciparum malaria [4]. In *P. falciparum* infections, it has been demonstrated that the shorter half-life and rapid clearance of red cells of G6PDd individuals make them less susceptible to malarial attacks from these parasites [133], and it is likely that a similar pathophysiological mechanism could occur in *P. vivax*-infected cells. However, there is a need to further explore and comprehend the mechanisms by which individuals with G6PDd are rendered less susceptible to infection. *P. vivax* preferentially invades reticulocytes, but *P. falciparum* can invade erythrocytes of all ages. This erythrocyte invasion is a significant phenomenon because G6PD activity is markedly reduced in older erythrocytes. Thus, red cells infected with *P. vivax* are most likely less vulnerable to the oxidant stress produced by the parasite. The precise mechanism by which G6PDd promotes reduced susceptibility to vivax malaria remains to be established.

## Concluding remarks

Currently, the only drug available for the elimination of *P. vivax* hypnozoites is PQ. Tafenoquine is another drug of the same group that is being evaluated in clinical trials [134]. This unique therapeutic class of drugs is extremely useful in the control and eventually, in the elimination of malaria. However, in the absence of a rapid diagnostic test to detect G6PDd, the potential for toxicity in individuals with this condition limits the safe and effective use of such drugs because of the hazardous and even life-threatening side effects. Indeed, this work shows that haemolysis in patients using PQ is not infrequent and contributes to the morbidity of infection caused by *P. vivax* in LA, thus representing the major clinical complication of G6PDd in this continent. PQ-induced haemolysis was only reported in males, thus indicating that testing only this population for G6PDd may be a more efficient and cheaper measure for minimising the risk of clinically relevant haemolysis after PQ administration in LA malaria endemic areas.

It is likely that the real impact of G6PDd in terms of malaria-related complications has been heavily underestimated, and further research should be devoted to clarifying the real burden that these complications impose on the health systems. Finally, this study highlights the need to improve current strategies for diagnosing and managing G6PDd in malaria field conditions. Malaria control programmes in LA need to take this condition into serious account in their national guidelines if measures such as massive PQ administration are considered as part of regional malaria elimination agendas.

## Abbreviations

AHA: Acute haemolytic anaemia; CHSA: Chronic non-sphaerocytic anaemia; G6PD: Glucose-6-phosphate dehydrogenase; G6PDd: Glucose-6-phosphate dehydrogenase deficiency; LA: Latin America; NNJ: Neonatal jaundice; PQ: Primaquine.

## Competing interests

MVGL was one of the principal investigators of the multicentre tafenoquine phase II trial, which is funded by GSK. The other authors declare that they have no competing interests.

## Authors’ contributions

WMM and MVGL conceived and designed the study. WMM and GPF performed a systematic review of the primary literature. WMM, GPF, ALMQ and GCM drafted the manuscript. MB, MRFO, HP, GASR. QB and MVGL directed the organisation and edited the manuscript. All authors read and approved the final version of the manuscript.

## Supplementary Material

Additional file 1 Summary of the findings organized by the type of stressor triggering haemolysis in G6PDd patients. Click here for file

## References

[B1] Luzzatto L Glucose 6-phosphate dehydrogenase deficiency: from genotype to phenotype Haematologica 2006 91 1303 1306 17018377

[B2] Cappellini MD Fiorelli G Glucose-6-phosphate dehydrogenase deficiency Lancet 2008 371 64 74 10.1016/S0140-6736(08)60073-2 18177777

[B3] Ruwende C Khoo SC Snow RW Yates SN Kwiatkowski D Gupta S Warn P Allsopp CE Gilbert SC Peschu N Natural selection of hemi- and heterozygotes for G6PD deficiency in Africa by resistance to severe malaria Nature 1995 376 246 249 10.1038/376246a0 7617034

[B4] Guindo A Fairhurst RM Doumbo OK Wellems TE Diallo DA X-linked G6PD deficiency protects hemizygous males but not heterozygous females against severe malaria PLoS Med 2007 4 e66 10.1371/journal.pmed.0040066 17355169 PMC1820604

[B5] Howes RE Piel FB Patil AP Nyangiri OA Gething PW Dewi M Hogg MM Battle KE Padilla CD Baird JK Hay SI G6PD deficiency prevalence and estimates of affected populations in malaria endemic countries: a geostatistical model-based map PLoS Med 2012 9 e1001339 10.1371/journal.pmed.1001339 23152723 PMC3496665

[B6] Beutler E Duparc S Glucose-6-phosphate dehydrogenase deficiency and antimalarial drug development Am J Trop Med Hyg 2007 77 779 789 17978087

[B7] White NJ The role of anti-malarial drugs in eliminating malaria Malar J 2008 7 S8 10.1186/1475-2875-7-S1-S8 19091042 PMC2604872

[B8] World Health Organization The Safety and Effectiveness of Single Dose Primaquine as a P. Falciparum Gametocytocide 2012 Geneva: World Health Organization

[B9] Nkhoma ET Poole C Vannappagari V Hall SA Beutler E The global prevalence of glucose-6-phosphate dehydrogenase deficiency: a systematic review and meta-analysis Blood Cells Mol Dis 2009 42 267 278 10.1016/j.bcmd.2008.12.005 19233695

[B10] Howes RE Dewi M Piel FB Monteiro WM Battle KE Messina JP Sakuntabhai A Satyagraha AW Williams TN Baird JK Spatial patterns of G6PD deficiency variants across malaria endemic regions Malar J 2013 12 418 10.1186/1475-2875-12-418 24228846 PMC3835423

[B11] Ruiz W Ulloa V Bailón O [Prevalence of glucose 6-phosphate dehydrogenase deficiency in the blood of voluntary givers at Cayetano Heredia and Arzobispo Loayza nationals hospitals Lima-Perú](in Spanish) Rev Méd Hered 1997 8 11 18

[B12] Barretto OCO [Erythrocyte glucose-6-phosphate dehydrogenase deficiency in Sao Paulo, Brazil](in Portuguese) Rev Bras Pesq Med Biol 1970 3 61 65

[B13] Sena LLA Ramalho AS [Clinical evaluation of glucose - 6 - phosphate dehydrogenase (G-6-PD) deficiency in a Brazilian population] (in Portuguese) Rev Bras Genét 1985 8 89 96

[B14] Sena LL Ramalho AS Barreto OC de Lima FA [Glucosephosphate dehydrogenase deficiency: data on prevalence and morbidity in the region of Natal, RN] (in Portuguese) AMB Rev Assoc Med Bras 1986 32 17 20 3491387

[B15] Kühn VL Lisbôa V de Cerqueira LP [Glucose-6-phosphate dehydrogenase deficiency in blood donors in a general hospital of Salvador, Bahia, Brazil] (in Portuguese) Rev Paul Med 1983 101 175 177 6672977

[B16] Azevêdo WC Silva ML Grassi MC Azevêdo ES [Glucose-6-phosphate dehydrogenase deficiency in a general hospital of Salvador, Bahia, Brazil] (in Portuguese) Rev Bras Pesq Med Biol 1978 11 49 52 653016

[B17] Castro SM Weber R Matte Ú Giugliani R Molecular characterization of glucose-6-phosphate dehydrogenase deficiency in patients from the southern Brazilian city of Porto Alegre, RS Genet Mol Biol 2007 30 10 13 10.1590/S1415-47572007000100003

[B18] Lisker R Pérez-Briceño R Beutler E A new glucose-6-phosphate dehydrogenase variant, Gd(−) Tepic, characterized by moderate enzyme deficiency and mild episodes of hemolytic anemia Hum Genet 1985 69 19 21 10.1007/BF00295523 3967887

[B19] Gutierrez A Garcia M Estrada M Quintero I Gonzalez R Glucose-6-phosphate dehydrogenase (G6PD) Guantanamo and G6PD Caujeri: two new glucose-6-phosphate dehydrogenase-deficient variants found in Cuba Biochem Genet 1987 25 231 238 10.1007/BF00499316 3606560

[B20] Weimer TA Schuler L Beutler E Salzano FM Gd (+) Laguna, a new rare glucose-6-phosphate dehydrogenase variant from Brazil Hum Genet 1984 65 402 404 10.1007/BF00291568 6693129

[B21] Saenz GF Chaves M Berrantes A Elizondo J Montero AG Yoshida A A glucose-6-phosphate dehydrogenase variant, Gd(−) Santamaria found in Costa Rica Acta Haematol 1984 72 37 40 10.1159/000206354 6433630

[B22] Lisker R Briceno RP Agrilar L Yoshida A A variant glucose-6-phosphate dehydrogenase Gd(−) Chiapas associated with moderate enzyme deficiency and occasional hemolytic anemia Hum Genet 1978 43 81 84 10.1007/BF00396481 669721

[B23] Lisker R Pérez-Briceño R Ravé V Yoshida A [Federal District glucose-6-phosphate dehydrogenase Gd(−). A new variant associated with moderate enzyme deficiency and occasional hemolytic anemia](in Portuguese) Rev Invest Clin 1981 33 209 211 7291768

[B24] Vaca G Ibarra B Hernandez A Velazquez AL Gonzalez-Quiroga G Romero F Medina C Zuniga P Martinez G Alvarez-Arratia MC Screening for inborn errors of the erythrocyte metabolism in Northwestern Mexico Acta Anthropogenet 1982 6 255 264 7187239

[B25] Aroca R Tomalá C Medrano A Holguín E [Most frequently causes of hemolytic anemia in children younger than 14 years. Roberto Gilbert Hospital of Guayaquil](in Spanish) Medicina 2005 10 267 270

[B26] Guzman C Etcheverry R Puga F Regonesi C Murabda M Duran N Munoz E [Hemolytic anemia caused by enzymatic defect (glucose-6-phosphate dehydrogenase deficiency). Research in Chilean populations: Mapuche, Pascuense and Alacalufe](in Spanish) Rev Med Chil 1964 92 592 600 14235178

[B27] Van der Sar A Schouten H Boudier AM Glucose-6-phosphate dehydrogenase deficiency in red cells. Incidence in the Curaçao population, its clinical and genetic aspects Enzymologia 1964 27 289 310 14228390

[B28] Pérez Vargas L Salas González C Haemolytic anaemia after nalidixic acid Lancet 1967 2 97 98 4165477 10.1016/s0140-6736(67)92084-3

[B29] Bloch M Sancho G Rivera H Characteristics of GPD deficiency in El Salvador Sangre (Barc) 1970 15 163 169 5464487

[B30] Barretto OC [New variant of erythrocyte glucose-6-phosphate dehydrogenase: Gd Sao Paulo](in Portuguese) Rev Hosp Clin 1983 38 247 248 6678007

[B31] Nunes AA Patient with toxoplasmosis and glucose-6-phosphate dehydrogenase deficiency: a case report Cases J 2009 2 8826 10.4076/1757-1626-2-8826 19918404 PMC2769474

[B32] McCurdy PR Maldonado N Dillon DE Conrad ME Variants of glucose-6-phosphate dehydrogenase (G-6-PD) associated with G-6-PD deficiency in Puerto Ricans J Lab Clin Med 1973 82 432 437 4728291

[B33] Beiguelman B Pinto W Jr Dall’aglio FF Da Silva E Vozza J [G-6-PD deficiency and leprosy](in Spanish) Cienc Cult 1966 18 95 96

[B34] Barraviera B Mendes RP Pereira PC Machado JM Curi PR Meira DA Measurement of glucose-6-phosphate dehydrogenase and glutathione reductase activity in patients with paracoccidioidomycosis treated with ketoconazole Mycopathologia 1988 104 87 91 3221915 10.1007/BF00436932

[B35] Abreu MP Freire CC Miura RS [Anesthesia in glucose 6-phosphate dehydrogenase-deficient patient: case report](in Portuguese) Rev Bras Anestesiol 2002 52 707 711 10.1590/S0034-70942002000600007 19475242

[B36] Howell SB Cook JA Treatment of schistosomiasis mansoni with hycanthone in glucose-6-phosphate dehydrogenase deficiency in St. Lucia Trans R Soc Trop Med Hyg 1971 65 331 333 10.1016/0035-9203(71)90008-3 5559749

[B37] Martínez Pérez JL Hadad Meléndez P [Primaquine-induced hemolytic syndrome and glucose 6 phosphate dehydrogenase deficiency](in Spanish) Rev Cuba Med Trop 1989 41 299 306 2486226

[B38] Chadee DD Tilluckdharry CC Doon R Imported cerebral malaria complicated with glucose-6-phosphate dehydrogenase deficiency West Indian Med J 1996 45 97 99 8952432

[B39] Menéndez Capote R Díaz Pérez L Luzardo Suárez C [Hemolysis and primaquine treatment](in Spanish) Rev Cubana Med Trop 1997 49 136 138 9685977

[B40] Silva MC Santos EB Costal EG Filho MG Guerreiro JF Povoa MM [Clinical and laboratorial alterations in *Plasmodium vivax* malaria patients and glucose-6-phosphate dehydrogenase deficiency treated with primaquine at 0.50 mg/kg/day](in Portuguese) Rev Soc Bras Med Trop 2004 37 215 217 10.1590/S0037-86822004000300004 15330059

[B41] Ramos Junior WM Sardinha JF Costa MR Santana MS Alecrim MG Lacerda MV Clinical aspects of hemolysis in patients with P. vivax malaria treated with primaquine, in the Brazilian Amazon Braz J Infect Dis 2010 14 410 412 20963329 10.1590/s1413-86702010000400017

[B42] Santana MS de Lacerda MVG Barbosa MGV Alecrim WD Alecrim MGC Glucose-6-phosphate dehydrogenase deficiency in an endemic area for malaria in Manaus: a cross-sectional survey in the Brazilian Amazon PLoS One 2009 4 e5259 10.1371/journal.pone.0005259 19370159 PMC2667256

[B43] Santana MS Monteiro WM Siqueira AM Costa MF Sampaio V Lacerda MV Alecrim MG Glucose-6-phosphate dehydrogenase deficient variants are associated with reduced susceptibility to malaria in the Brazilian Amazon Trans R Soc Trop Med Hyg 2013 107 301 306 10.1093/trstmh/trt015 23479361

[B44] Lacerda MVG Fragoso SCP Alecrim MGC Alexandre MAA Magalhães BML Siqueira AM Ferreira LCL Araújo JR Mourão MPG Ferrer M Castillo P Martin-Jaular L Fernandez-Becerra C del Portillo H Ordi J Alonso PL Bassat Q Postmortem characterization of patients with clinical diagnosis of *Plasmodium vivax* malaria: to what extent does this parasite kill? Clin Infect Dis 2012 55 e67 e74 10.1093/cid/cis615 22772803

[B45] Acosta T Suárez M Núñes V Marín LC Cordero A [Hemolytic effect of chloroquine in students with glucose-6-phosphate dehydrogenase deficiency] (in Spanish) Rev Cuba Invest Bioméd 2003 22 180 185

[B46] Stekel A Rozovski J Saelzer E [Favism Report of two cases in Chile] (in Spanish) Rev Chil Pediatr 1973 44 265

[B47] Rojas J Dujisin K Calvo C [Clinical study of three cases of favism] (in Spanish) Rev Méd Chile 1982 110 1219 1222 7184110

[B48] Torres CD Chandía CM [Favism presenting as an acute renal failure: report of one case] (in Spanish) Rev Med Chil 2012 140 1043 1045 10.4067/S0034-98872012000800011 23282778

[B49] González G Henríquez P Delgado C Araya C Pereira J [Hemolitic anemia due to fava bean consumption] (in Spanish) Pediatr Día 2006 22 33 35

[B50] Eandi-Eberle S García Rosolen N Urtasun C Sciuccati G Díaz L Savietto V Candás A Avalos Gómez V Cervio C Bonduel M Feliú Torres A [Glucose 6 phosphate dehydrogenase deficiency: a case series] (in Spanish) Arch Argent Pediatr 2011 109 354 356 10.5546/aap.2011.354 21829878

[B51] Rivero MEJ Diniz EMA Nonoyama K Barretto OCO Vaz FAC [Deficiency of glucose-6-phosphate dehydrogenase in newborns] (in Spanish) Pediatr 1981 3 214 216

[B52] Vaca G Ibarra B Hernández A Olivares N Medina C Sánchez-Corona J Wunsch C Godínez B Martínez-Basalo C Cantú JM Glucose-6-phosphate dehydrogenase deficiency and abnormal hemoglobins in Mexican newborns with jaundice Rev Invest Clin 1981 33 259 261 7330495

[B53] Silva AS Oliveira LS Costa GN Lima GMS Carvalho TCR [Deficiency of glucose-6-dehydrogenases phosphate: frequecy, laboratorial and clinic aspects](in Portuguese) Rev IMIP 1991 5 113 116

[B54] Iglessias MAC Santos RMV Amorim MST Silva RT Moreira SS Barretto OCO Medeiros TMD [Erythrocyte glucose-6-phosphate dehydrogenase deficiency in male newborn babies and its relationship with neonatal jaundice](in Portuguese) Rev Bras Hematol Hemoter 2010 32 434 438 10.1590/S1516-84842010005000086

[B55] Torregrosa MV Neonatal jaundice in Puerto Rico (hemolytic disease) Bol Asoc Med P R 1970 62 141 146 5269145

[B56] Ramalho AS [Deficiency of gluscose 6-phosphate dehydrogenase (G-6-PD) in Brazilian newborns](in Portuguese) F Méd 1980 81 603 606

[B57] Henny-Harry C Trotman H Epidemiology of neonatal jaundice at the University Hospital of the West Indies West Indian Med J 2012 61 37 42 22808564

[B58] Boada Boada JJ [Glucose-6-phosphate-dehydrogenase: incidence and importance in neonatal jaundice](in Spanish) Acta Cient Venez 1967 18 41 43 4973011

[B59] Chaves M Quintana E Sáenz GF Monge G Aguero O Montero A Jiménez J Neonatal icterus and erythrocyte glucose-6-phosphate dehydrogenase deficiency. Experience in Costa Rica Sangre (Barc) 1987 32 428 435 3660180

[B60] Gibbs WN Gray R Lowry M Glucose-6-phosphate dehydrogenase deficiency and neonatal jaundice in Jamaica Br J Haematol 1979 43 263 274 10.1111/j.1365-2141.1979.tb03750.x 508636

[B61] Gurrola GC Araúz JJ Durán E Aguilar-Medina M Ramos-Payán R García-Magallanes N Pacheco GV Meraz EA Kernicterus by glucose-6-phosphate dehydrogenase deficiency: a case report and review of the literature J Med Case Rep 2008 2 146 10.1186/1752-1947-2-146 18460213 PMC2391151

[B62] Verdy E Herve J Boisson C Combrisson A Can glucose-6-phosphate dehydrogenase deficiency alone explain neonatal jaundice Rev Fr Transfus Immunohematol 1978 21 1081 1091 10.1016/S0338-4535(78)80005-1 754244

[B63] Azevedo ES Azevedo TFS [Glucose-6-phosphate dehydrogenase deficiency and neonatal jaundice in Bahia, Brazil](in Portuguese) Cienc Cult 1974 26 44 47

[B64] Perkins RP The significance of glucose-6-phosphate dehydrogenase deficiency in pregnancy Am J Obstet Gynecol 1976 125 215 223 817603 10.1016/0002-9378(76)90596-2

[B65] Paixäo AC Gonçalves AL Borges EG Tone LG [Screening test for glucose-6-phosphate dehydrogenase deficiency](in Portuguese) Rev Bras Patol Clín 1986 22 118 121

[B66] Velázquez AL Rico NG Ibarra B Blancarte R Cardosa J Fonseca S Maldonado E Enríquez MA Medina C Cantú JM Vaca G Hereditary erythroenzymopathies in neonates with hyperbilirubinemia Bol Méd Hosp Infant Méx 1985 42 466 469 4052226

[B67] González González OL Hidalgo Calcines PC Erythrocytary metabolic disorders in term newborn infants with physiological jaundice, dicreased glutation and glucose-6-phosphate-dehydrogenase Medicentro 1986 2 89 93

[B68] Sutton RN Erythrocyte glucose-6-phosphate-dehydrogenase deficiency in Trinidad Lancet 1963 1 855 13979427 10.1016/s0140-6736(63)91626-x

[B69] Mezzacappa MA Facchini FP Pinto AC Cassone AEL Souza DS Bezerra MAC Albuquerque DM Saad STO Costa FF Clinical and genetic risk factors for moderate hyperbilirubinemia in Brazilian newborn infants J Perinatol 2010 30 819 826 10.1038/jp.2010.48 20376058

[B70] Carvalho CG Castro SM Santin AP Zaleski C Carvalho FG Giugliani R Glucose-6-phosphate-dehydrogenase deficiency and its correlation with other risk factors in jaundiced newborns in Southern Brazil Asian Pac J Trop Biomed 2011 1 110 113 23569738 10.1016/S2221-1691(11)60006-3 PMC3609178

[B71] Gonzalez-Quiroga G del Rio JL R Ortiz-Jalomo R Garcia-Contreras RF Cerda-Flores RM Mata-Cardenas BD Garza-Chapa R [Relative frequency of glucose-6-phosphate dehydrogenase deficiency in jaundiced newborn infants in the metropolitan area of Monterrey, Nuevo Leon](in Spanish) Arch Invest Med 1990 21 223 227 2131769

[B72] González-Quiroga G Ramirez-Del Rio JL Cerda-Flores RM Garza-Chapa R Frequency and origin of G-6-PD deficiency among icteric newborns in the metropolitan area of Monterrey, Nuevo León, Mexico Gene Geogr 1994 8 157 164 7662606

[B73] Garlipp CR Ramalho AS [Clinical and laboratory aspects of glucose-6-phosphate dehydrogenase (G-6-PD) deficiency in Brazilian newborns](in Portuguese) Rev Bras Genet 1988 11 717 728

[B74] Iglessias MAC Frequency of glucose-6-phosphate dehydrogenase deficiency (G-6-PD) and its relationship with neonatal jaundice Rev Bras Hematol Hemoter 2009 31 57

[B75] Estrada M Gonzalez R [Neonatal jaundice and glucose-6-phosphate dehydrogenase deficiency in Havana] (in Spanish) Rev Invest Clin 1983 35 297 299 6672927

[B76] Alencastro de Azevedo L Reverbel da Silveira T Carvalho CG Martins de Castro S Giugliani R Matte U UGT1A1, SLCO1B1, and SLCO1B3 polymorphisms vs. neonatal hyperbilirubinemia: is there an association? Pediatr Res 2012 72 169 173 10.1038/pr.2012.60 22580719

[B77] Trotman H Henny-Harry C Factors associated with extreme hyperbilirubinaemia in neonates at the University Hospital of the West Indies Paediatr Int Child Health 2012 32 97 101 10.1179/2046905512Y.0000000014 22595218

[B78] Saad ST Salles TS Carvalho MH Costa FF Molecular characterization of glucose-6-phosphate dehydrogenase deficiency in Brazil Hum Hered 1997 47 17 21 10.1159/000154383 9017974

[B79] Barretto OC Nonoyama K Gd(−) Carapicuiba, a rare glucose-6-phosphate dehydrogenase variant associated with moderate enzyme deficiency and chronic hemolysis Braz J Med Biol Res 1991 24 133 139 1823224

[B80] Dal Borgo AP Rosario SC Cavieres AM Dos nuevas mutaciones de glucosa 6 fosfato deshidrogenasa, G6PD Santiago y G6PD Calvo Mackenna Revista Chil Pediatr 2000 71 419 422

[B81] Estrada M García M Gutierrez A Quintero I Gonzalez R G6PD Varadero. A new variant of glucose-6-phosphate dehydrogenase associated with congenital nonspherocytic hemolytic anemia Vox Sang 1982 43 102 104 10.1111/j.1423-0410.1982.tb00533.x 7123903

[B82] Elizondo J Sáenz GF Páez CA Ramón M García M Gutiérrez A Estrada M G6PD-Puerto Limón: a new deficient variant of glucose-6-phosphate dehydrogenase associated with congenital nonspherocytic hemolytic anemia Hum Genet 1982 62 110 112 7160841 10.1007/BF00282295

[B83] Zilberstein B Eshkenazy R Ribeiro Júnior MA Sallet JA Ramos AC Laparoscopic cholecystectomy in children and adolescents Sao Paulo Med J 1996 114 1293 1297 10.1590/S1516-31801996000600002 9269102

[B84] Lírio AS López KC Bernardo MA [Glucose-6-phosphate dehydrogenase among blood donors of the State of Rio de Janeiro] (in Portuguese) Folha Médica 1980 80 705 707

[B85] Sánchez MC Villegas VE Fonseca D [Glucose-6-phosphate dehydrogenase deficiency: enzimatic and molecular analysis in a Bogotá population] (in Spanish) Colomb Med 2008 39 14 23

[B86] Severo LG Nogueira DM Hoxter G [Determination of the glucose-6-phosphate (G-6-PD) activity in human erythrocytes] (in Spanish) Laes & Haes 1985 13 22 30

[B87] Medeiros TMD Abreu A Albuquerque LMM Lins MRS Abnormal hemoglobins and erythrocyte glucose-6- phosphate dehydrogenase deficiency in Natal, RN Rev Bras Patol Clín 1992 28 43 47

[B88] Saad ST Costa FF Glucose-6-phosphate dehydrogenase deficiency and sickle cell disease in Brazil Hum Hered 1992 42 125 128 10.1159/000154052 1572671

[B89] Katsuragawa TH Cunha RPA de Souza DCA Gil LHS Cruz RB Silva AAE Tada MS da Silva LHP [Malaria and hematological aspects among residents to be impacted by reservoirs for the Santo Antônio and Jirau Hydroelectric Power Stations, Rondônia State, Brazil](in Portuguese) Cad Saude Publica 2009 25 1486 1492 10.1590/S0102-311X2009000700006 19578569

[B90] Cardoso MA Scopel KKG Muniz PT Villamor E Ferreira MU Underlying factors associated with anemia in Amazonian children: a population-based, cross-sectional study PLoS One 2012 7 e36341 10.1371/journal.pone.0036341 22574149 PMC3344855

[B91] Roberts DF Triger DR Morgan RJ Glucose-6-phosphate dehydrogenase deficiency and haemoglobin level in Jamaican children West Indian Med J 1970 19 204 211 5504403

[B92] del Luján AI Milani AC Pérez SM Lanza O Detarsio G Erythrocyte glucose-6-phosphate dehydrogenase deficiency in Rosario Acta Bioquim Clin Latinoam 2012 46 359 363

[B93] Nicolielo DB Ferreira RIP Leite AA Activity of 6-phosphogluconate dehydrogenase in glucose-6-phosphate dehydrogenase deficiency Rev Bras Hematol Hemoter 2006 28 135 138

[B94] de Miani MS A Peñalver JA Incidence of beta-thalassemia carriers and those deficient in erythrocyte glucose-6-phosphate dehydrogenase in the greater Buenos Aires area Sangre (Barc) 1983 28 537 541 6665690

[B95] Azevêdo ES Alves AF Da Silva MC Souza MG Muniz Dias Lima AM Azevedo WC Distribution of abnormal hemoglobins and glucose-6-phosphate dehydrogenase variants in 1200 school children of Bahia, Brazil Am J Phys Anthropol 1980 53 509 512 10.1002/ajpa.1330530407 7468787

[B96] Madrigal L Sáenz G Chávez M Glucose-6-phosphate dehydrogenase deficiency: its frequency in Hb AS and Hb AA individuals among the black population of Limón Sangre (Barc) 1990 35 413 414 2291155

[B97] Sáenz BR Jiménez DM Chaves VM Quintana GEM Sáenz RGF Coexistence of hemoglobin S and glucose-6-phosphate dehydrogenase deficiency in negroid population Rev Costarric Cienc Méd 1986 7 305 310

[B98] Miall WE Milner PF Lovell HG Standard KL Haematological investigations of population samples in Jamaica Br J Prev Soc Med 1967 21 45 55 6034386 10.1136/jech.21.2.45 PMC1059071

[B99] Lewgoy F Salzano FM [Dynamics of the gene that determines the deficiency in G-6-PD in the population of Porto Alegre](in Portuguese) Cienc Cult 1965 2 152

[B100] Kahn A Boivin P Lagneau J Phenotypes of erythrocytic glucose-6-phosphate dehydrogenase in black people. Examination of 301 black people living in France and description of 9 different variants. High incidence of deficiency of an enzyme of “B” mobility Humangenetik 1973 18 261 270 4719636 10.1007/BF00290606

[B101] Gibbs WN Ottey F Dyer H Distribution of glucose-6-phosphate dehydrogenase phenotypes in Jamaica Am J Hum Genet 1972 24 18 23 5012688 PMC1762152

[B102] Salzano FM Lewgoy F Tondo CV da Rocha FJ G-6-PD deficiency and abnormal hemoglobins in a Brazilian population Acta Genet Med Gemellol (Roma) 1968 17 607 612 5732547 10.1017/s1120962300012476

[B103] Saad ST Costa FF Salles TS Sonatti MF Figueiredo MS Glucose-6-phosphate dehydrogenase deficiency in sickle cell disease by DNA analysis Blood 1995 85 601 602 7812018

[B104] Gibbs WN Wardle J Serjeant GR Glucose-6-phosphate dehydrogenase deficiency and homozygous sickle cell disease in Jamaica Br J Haematol 1980 45 73 80 10.1111/j.1365-2141.1980.tb03812.x 7378331

[B105] Castro de Kolster C Rolo M Arias S Guerreiro N Carvajal A Castro J Kolster J Hepatic glycogenosis: the clinical, biochemical and enzymatic aspects in a group of pediatric patients G E N 1992 46 191 198 1340824

[B106] Itskan SB Saldanha PH [Erythrocyte glucose-6-phosphate dehydrogenase activity in the population of a malarial region in Sao Paulo (Iguape)](in Portuguese) Rev Inst Med Trop Sao Paulo 1975 17 83 91 1153914

[B107] Barraviera B Meira DA Machado PEA Curi PR Malaria in the municipality of Humaitá state of Amazonas: XXI. Prevalence of glucose-6 phosphate dehidrogenase in a population sample and in patients with malaria caused by *Plasmodium falciparum* Rev Inst Med Trop Sao Paulo 1987 29 374 380 10.1590/S0036-46651987000600007 3331490

[B108] Beiguelman B Alves FP Moura MM Engracia V Nunes AC Heckmann MI Ferreira RG da Silva LH Camargo EP Krieger H The association of genetic markers and malaria infection in the Brazilian Western Amazonian region Mem Inst Oswaldo Cruz 2003 98 455 460 10.1590/S0074-02762003000400004 12937753

[B109] Guevara A Calvopiña M Macías G Guderian R Deficiencia de glucosa-6-fosfato dehidrogenasa en poblaciones ecuatorianas de raza negra Acta Bioquím Clín Latinoam 1991 25 113 117

[B110] Moyano M Méndez F Erythrocyte defects and parasitemia density in patients with *Plasmodium falciparum* malaria in Buenaventura, Colombia Rev Panam Salud Publica 2005 18 25 32 10.1590/S1020-49892005000600005 16105323

[B111] Carmona-Fonseca J Álvarez G Ríos A Vásquez MF Deficiency of glucose-6-phosphate dehydrogenase in healthy men and malaria patients; Turbo (Antioquia, Colombia) Rev Bras Epidemiol 2008 11 252 265 10.1590/S1415-790X2008000200007

[B112] Monte Alegre S Saad ST Delatre E Saad MJ Insulin secretion in patients deficient in glucose-6-phosphate dehydrogenase Horm Metab Res 1991 23 171 173 10.1055/s-2007-1003644 1874475

[B113] Vaca G Ramirez MG Vargas M Mendoza R Chavez-Anaya E Medina MD Alvarez A Medina C Saenz G Chavez M Effects of G-6-PD deficiency, experimentally induced or genetically transmitted, on the sorbitol pathway activity. *In vitro* and *in vivo* studies Arch Med Res 1992 23 25 32 1308788

[B114] Saad MJ Monte-Alegre S Saad ST Cortisol levels in glucose-6-phosphate dehydrogenase deficiency Horm Res 1991 35 1 3 1655604 10.1159/000181866

[B115] Santana MS da Rocha MA Arcanjo AR Sardinha JF Alecrim WD Alecrim M [Association of methemoglobinemia and glucose-6-phosphate dehydrogenase deficiency in malaria patients treated with primaquine] (in Portuguese) Rev Soc Bras Med Trop 2007 40 533 536 10.1590/S0037-86822007000500008 17992408

[B116] Ferreira MES Gomes MSM Vieira JLF Methemoglobinemia in patients with *Plasmodium vivax* receiving oral therapy with primaquine Rev Soc Bras Med Trop 2011 44 113 115 10.1590/S0037-86822011000100026 21340422

[B117] Bonilla JF Palomino F [Exercise-induced hemolysis: relation between the activity of glucose-6-phosphate dehydrogenase and the magnitude of the hemolysis] (in Spanish) Colomb Med 2008 39 126 134

[B118] Barretto OC Cardoso JL De Cillo D Viscerocutaneous form of loxoscelism and erythrocyte glucose-6-phosphate deficiency Rev Inst Med Trop Sao Paulo 1985 27 264 267 10.1590/S0036-46651985000500006 3834578

[B119] Rosa-Borges A Sampaio MG Condino-Neto A Barreto OC Nudelman V Carneiro-Sampaio MM Nogueira SA Abreu TF Rehder J Costa-Carvalho BT [Glucose-6-phosphate dehydrogenase deficiency with recurrent infections: case report](in Portuguese) J Pediatr 2001 77 331 336 10.2223/jped.243 14647867

[B120] Agudelo-Florez P Costa-Carvalho BT Lopez JA Redher J Newburger PE Olalla-Saad ST Condino-Neto A Association of glucose-6-phosphate dehydrogenase deficiency and X-linked chronic granulomatous disease in a child with anemia and recurrent infections Am J Hematol 2004 75 151 156 10.1002/ajh.10477 14978696

[B121] Ramalho AS Minor thalassemia, sickle-cell trait and G6-PD deficiency: prevalence and morbility in the region of Campinas, SP Bol Soc Bras Hematol Hemoter 1985 7 133 136

[B122] Weimer TA Tavares Neto J Franco MHLP Hutz MH Salzano FM Kubo RR Rosa RTD Friedrisch JR Prata A Genetic aspects of *Schistosoma mansoni* infection severity Rev Bras Genét 1991 14 623 630

[B123] Beiguelman B Pinto W Jr Dall’aglio FF Da Silva E Vozza JA G-6PD deficiencyamong lepers and healthy people in Brazil Acta Genet Stat Med 1968 18 159 162 5694903

[B124] Guerra CA Howes RE Patil AP Gething PW Van Boeckel TP Temperley WH Kabaria CW Tatem AJ Manh BH Elyazar IR Baird JK Snow RW Hay SI The international limits and population at risk of *Plasmodium vivax* transmission in 2009 PLoS Negl Trop Dis 2010 4 e774 10.1371/journal.pntd.0000774 20689816 PMC2914753

[B125] World Health Organization WHO Malaria Report 2012 Geneva: World Health Organization

[B126] Vaca G Ibarra B Romero F Olivares N Cantú JM Beutler E G-6-PD Guadalajara. A new mutant associated with chronic nonspherocytic hemolytic anemia Hum Genet 1982 61 175 176 10.1007/BF00274214 7129446

[B127] Sáenz GF Chaves M Grant S Barrenechea M Arroyo G Valenciano E Jiménez J Montero AG Abnormal hemoglobins, alpha thalassemia and erythrocyte G6PD deficiency in newborn infants of the negroid race Sangre (Barc) 1984 29 861 867 6523338

[B128] Luzzatto L Warrell D, Cox TM, Firth JD Glucose 6-phosphate dehydrogenase deficiency Oxford Textbook of Medicine 2010 Oxford: Oxford University Press 4474 4479

[B129] Luzzatto L Allan NC Relationship between the genes for glucose-6-phosphate dehydrogenase and for haemoglobin in a Nigerian population Nature 1968 219 1041 1042 10.1038/2191041a0 5673357

[B130] Steinberg MH West MS Gallagher D Mentzer W Effects of glucose-6-phosphate dehydrogenase deficiency upon sickle cell anemia Blood 1988 71 748 752 3345344

[B131] Bouanga JC Mouele R Prehu C Wajcman H Feingold J Galacteros F Glucose-6-phosphate dehydrogenase deficiency and homozygous sickle cell disease in Congo Hum Hered 1998 48 192 197 10.1159/000022801 9694250

[B132] Piomelli S Siniscalco M The haematological effects of glucose-6-phosphate dehydrogenase deficiency and thalassaemia trait: interaction between the two genes at the phenotype level Br J Haematol 1969 16 537 549 10.1111/j.1365-2141.1969.tb00435.x 5802491

[B133] Cappadoro M Giribaldi G O’Brien E Turrini F Mannu F Ulliers D Simula G Luzzatto L Arese P Early phagocytosis of glucose-6-phosphate dehydrogenase (G6PD)-deficient erythrocytes parasitized by *Plasmodium falciparum* may explain malaria protection in G6PD deficiency Blood 1998 92 2527 2534 9746794

[B134] Llanos-Cuentas A Lacerda MV Rueangweerayut R Krudsood S Gupta SK Kochar SK Arthur P Chuenchom N Mohrle JJ Duparc S Ugwuegbulam C Kleim JP Carter N Green JA Kellam L Tafenoquine plus chloroquine for the treatment and relapse prevention of *Plasmodium vivax* malaria (DETECTIVE): a multicentre, double-blind, randomised, phase 2b dose-selection study Lancet 2013 S0140–6736 62568 4 10.1016/S0140-6736(13)62568-4 24360369

