# Supplementary material for: Clinical complications of G6PD deficiency in Latin American and Caribbean populations: systematic review and implications for malaria elimination programmes
Source: Malar J. 2014 Feb 25;13:70. doi: 10.1186/1475-2875-13-70 (PMC3938641; doi:10.1186/1475-2875-13-70)
Supplement: Additional file 1 — Summary of the findings organized by the type of stressor triggering haemolysis in G6PDd patients. [file 1475-2875-13-70-S1.docx]

**Additional File 1.** Summary of the findings organized by the type of stressor triggering haemolysis in G6PDd patients.

| **Country (Location)** | **Type of study** | **Year** | **Sample size** | **Study population** | **Haemolysis trigger** | **Major clinical findings** | **Reference** |
| --- | --- | --- | --- | --- | --- | --- | --- |
| **DRUG-INDUCED HAEMOLYSIS** | | | |  |  |  |  |
| Brazil (Campinas) | Case report | 2002 | 1 | Male patient, 9 years old | - | Spinal anesthesia with bupivacaine associated to total intravenous anesthesia with propofol has shown to be safe for G6PD-deficient patients | Abreu et al. (2002) |
| Cuba (La Habana) | Case report/series | 2003 | 8 | Students aged between 17 and 24 years old presenting imported malaria | - | Chloroquine treatment did not triggered hemolysis in 8 G6PD deficient subjects | Acosta-Sánchez et al. (2003) |
| Ecuador (Guayaquil) | Cross-sectional | 2005 | 6.736 | Children from the general population | Not available | 3.41/1000 cases of acute haemolytic anaemia. Frequency of G6PDd was 8.6% among 23 childrem with acute haemolytic anaemia | Aroca et al. (2005) |
| Brazil (Salvador) | Cross-sectional | 1978 | 792 | Male negroid population | Not available | Higher frequency of previous history of jaundice in G6PD-deficients (22,2%; n=63) in relation to non-G6PD deficients (12,8%; n=729) (RP=1,7, p=0,03);  G6PDd was not severe enough to require hospitalization | Azevedo et al. (1978) |
| Brazil (São Paulo) | Case report | 1988 | 38 | Patients with paracoccidioidomycosis | Ketoconazole | Hemoglobin rates, hematocrit and G6PD activity were measured in 38 patients with paracoccidioidomycosis treated with ketoconazole or sulfadoxin. Ketoconazole-treated patients showed reduced G6PD activity. One of these patients was found to be G6PD-deficient and suffered a hemolytic episode during treatment | Barraviera et al. (1988) |
| Brazil (São Paulo) | Cross-sectional | 1970 | 776 | General population | - | Absence of history of jaundice among 17 G6PD-deficient identified | Barreto (1970) |
| Brazil (São Paulo) | Case report | 1983 | 1 | Male patient, 3 years-old | Sulphadiazine | Acute haemolytic episode related to G6PDd with jaundice and severe anemia (Hemoglobin=4g/dL) after sulphadiazine use | Barretto (1983) |
| Brazil (Bauru) | Case series | 1966 | 17 | Patients with leprosy | - | Absence of acute haemolytic anaemia related to G6PDd in patients with leprosy under sulphone treatment | Beiguelman et al. (1966) |
| El Salvador (San Salvador) | Case report/series | 1970 | 4 | Patients presenting acute haemolytic anaemia | Primaquine (n=3);  Aspirin (n=1) | Acute haemolytic episodes related to G6PDd, needing red cell transfusion in 1 case of primaquine-induzed haemolysis | Bloch et al. (1970) |
| Brazil (Porto Alegre) | Cross-sectional | 2007 | 348 | Patients that presented acute haemolytic crises | - | Higher prevalence of G6PDd (A- variant; 10.3%) in patients with history of acute haemolytic crises in comparison with the general population | Castro et al. (2007) |
| Trinidad and Tobago (Port of Spain) | Case report | 1996 | 1 | Patient presenting cerebral falciparum malaria | Primaquine | G6PD-deficient patient with anaemia (needing RBC transfusion), blackwater fever, renal impairment | Chadee et al. (1996) |
| Chile (Santiago) | Case series | 1964 | 38 | Patients diagnosed with acute haemolytic anaemia | Not available (n=7);  Chloramphenicol (n=3);  Sulphadiazine (n=2);  Aspirin (n=2);  Acetaminosalol (n=1) | G6PDd in 39.5% of the patients (n=15), with some severe cases, but no detailed clinical description | Guzmán et al. (1964) |
| Saint Lucia (Castries) | Case report/series | 1971 | 8 | G6PD-deficient infected with *Schistosoma mansoni* | - | A single intramuscular dose (3 mg/kg body weight) of hycanthone. Serial haematocrit and reticulocyte counts showed no evidence of haemolysis related to G6PDd | Howell and Cook (1971) |
| Brazil (Salvador) | Case report/series | 1983 | 6 | Blood recipients | - | No alterations suggestive of haemolysis in patients who received blood donated by G6PD deficients, although some were using drugs able to trigger this complication | Kuhn et al. (1983) |
| Brazil (Manaus) | Case series | 2012 | 17 | *P. vivax***–**infected deceased patients | Primaquine (n=2) | In 2 cases the cause of death was directly related to hemolysis  triggered by primaquine in the presence of G6PDd. Both presented severe anemia, jaundice, acute renal failure, respiratory distress and neurological symptoms | Lacerda et al. (2012) |
| Mexico (Chiapas) | Case report | 1978 | 1 | A 36-years-old man | Not available | Occasional hemolytic anemia related to G6PDd | Lisker et al. (1978) |
| Mexico (Distrito Federal) | Case report | 1981 | 1 | A 1,5-year-old boy | Not available | Occasional hemolytic anemia related to G6PDd | Lisker et al. (1981) |
| Mexico (Tepic) | Case report | 1985 | 1 | A 16-years-old boy | Not available | Occasional hemolytic anemia related to G6PDd | Lisker et al. (1985) |
| Cuba (La Habana) | Cross-sectional | 1989 | 500 | Travelers returning to Cuba from endemic malarial areas | Primaquine (n=12) | 16 G6PD deficient patients identified from a group of 500, Haemolytic anaemia occurred in 12/16 patients (87.5%) between the fifth and seventh day of treatment | Martínez-Perez and Hadad-Meléndez (1989) |
| Puerto Rico (San José) | Case report/series | 1973 | 9 | Patients with history of haemolytic anaemia and their relatives | Primaquine (n=1);  Primaquine + sulfazoxazole + nitrofurantoin (n=1) | Two cases of acute haemolytic anaemia related to G6PDd due to primaquine alone or in combined treatments | McCurdy et al. (1973) |
| Cuba (La Habana) | Case report/series | 1997 | 8 | G6PD deficient patients with *P. vivax* malaria | Primaquine (n=6) | It was determined that 87.5% of the patients presented hemolysis. A half of the patients could not finish their treatment because of the appearance of important hemolysis | Menéndez-Capote et al. (1997) |
| Brazil (Ribeirão Preto) | Case report | 2009 | 1 | 5-years-old boy with toxoplasmosis | Sulfadiazine | Acute haemolytic anaemia related to G6PDd on the seventh day of treatment | Nunes (2009) |
| Chile (Santiago) | Case report | 1967 | 1 | 18-month-old boy | Nalidixic acid | Hemolytic episode related to G6PDd after nalidixic acid use | Pérez-Vargas and Salas-González (1967) |
| Brazil (Manaus) | Case series | 2010 | 18 | Patients with *P. vivax* malaria | Primaquine (n=18) | Haemolysis related to G6PDd accompanied by fever and leukocytosis, in addition to anemia requiring red blood cell transfusion, and development of acute renal failure | Ramos-Junior et al. (2010) |
| Peru (Lima) | Cross-sectional | 1997 | 140 | Male population | - | Absence of history of haemolysis for the only G6PD-deficient identified | Ruiz et al. (1997) |
| Costa Rica (San José) | Case report | 1984 | 1 | Adult man | Not available | Occasional hemolytic anemia related to G6PDd | Sáenz et al. (1984) |
| Brazil (Manaus) | Cross-sectional | 2009 | 200 | Male population | Not available | G6PDd was associated with a history of jaundice (OR=11.9 95%IC 1.3-78.3; p=0.012) and need of blood transfusion (p<0.001) during previous treatment for malarial infection | Santana et al. (2009) |
| Brazil (Manaus) | Cross-sectional | 2013 | 1,478 | Male population | Not available | G6PDd was associated with a history of blood transfusion (p<0.001) during previous treatment for malarial infection | Santana et al. (2013) |
| Brazil (Campinas) | Cross-sectional | 1985 | 3,339 | Male population | - | Absence of association of history of haemolysis and the presence of G6PDd (n=66) | Sena and Ramalho (1985) |
| Brazil (Natal) | Cross-sectional | 1986 | 719 | Male population | - | Absence of association of history of haemolysis and the presence of G6PDd (n=19) | Sena et al. (1986) |
| Brazil (Belém) | Case report/series | 2004 | 3 | G6PD deficient patients with *P. vivax* malaria | Primaquine (n=3) | Acute haemolytic anaemia related to G6PDd, requiring treatment discontinuation | Silva et al. (2004) |
| Mexico (Guadalajara) | Case series | 1982 | 54 | Patients with haemolytic anaemia | Not available | The frequency of G6PDd was 30.3% | Vaca et al. (1982) |
| Curaçao (Willemstad) | Case report/series | 1964 | 4 | Patients with haemolytic anaemia | Naphtalene (n=1);  Rum (n=1);  Wine (n=1);  Salycilates (n=1);  Dapsone (n=1) | Haemolytic anaemia in G6PDd patients related to naphthalene intoxication, after excessive intake of rum and wine and after salycilates and dapsone treatment (in a G6PD deficient recipient with leprosy, under dapsone therapy).  Combining blood transfusion and ACTH therapy, haemolytic anaemia had a benign course | Van der Sar et al. (1964) |
| Brazil (Laguna) | Case report | 1984 | 1 | 9-year-old boy | Not available | Acute haemolytic anaemia related to G6PDd | Weimer et al. (1984) |
| **FAVISM** |  |  |  |  |  |  |  |
| Chile (Santiago) | Case report | 1964 | 2 | Two boys (age not available) | *Vicia faba* | Acute haemolytic anaemia related to G6PDd | Guzmán et al. (1964) |
| Chile (Santiago) | Case report | 1973 | 2 | Two boys (age not available) | *Vicia faba* | Acute haemolytic anaemia related to G6PDd | Stekel et al. (1973) |
| Chile (Chillán, Curanilahue, and Los Angeles) | Case report | 1982 | 3 | Two 14-year-old men and one 8-year-old boy | *Vicia faba* | Acute haemolytic anaemia related to G6PDd, needing for red cell transfusion in two cases | Rojas et al. (1982) |
| Chile (Santiago) | Case report | 2006 | 1 | 2-year-old boy | *Vicia faba* | Acute haemolytic anaemia related to G6PDd, without need for red cell transfusion | González et al. (2006) |
| Chile (Santiago) | Case report | 2012 | 1 | 67-year-old man | *Vicia faba* | Acute haemolytic anaemia related to G6PDd (needing for red cell transfusion) evolving to acute renal failure) | Torres and Chandía (2012) |
| **INFECTION-INDUCED HAEMOLYSIS** | | | |  |  |  |  |
| Curaçao (Willemstad) | Case report | 1964 | 1 | 50-year-old man | Infection (unknown aetiological agent; patient using chloramphenicol) | Mild acute haemolytic anaemia with jaundice, related to G6PDd | Van der Sar (1964) |
| El Salvador (San Salvador) | Case report | 1970 | 1 | 2-years-old boy | Infection (unknown aetiological agent; patient using aspirin) (n=01) | Acute haemolytic anaemia related to G6PDd (required two exchange transfusions) | Bloch et al. (1970) |
| Cuba (Guantánamo and Caujerí) | Case report | 1987 | 1 | 8-year-old boy | Recurrent viral and bacterial infections | Acute haemolytic anaemia related to G6PDd | Gutierrez et al. (1987) |
